# Core biopsy as a tool in planning the management of invasive breast cancer

**DOI:** 10.1186/1477-7819-3-1

**Published:** 2005-01-04

**Authors:** Amar Deshpande, Trivikram Garud, Simon D Holt

**Affiliations:** 1 Department of General Surgery, University Hospital, Birmingham, UK; 2 Department of General Surgery, North Tees University Hospital, Stockton-on-Tees, UK; 3 Department of Surgery, Prince Phillip Hospital, Llanelli, UK

## Abstract

**Background:**

Core biopsy is a method of choice for the triple assessment of breast disease as it can reliably distinguish between benign and malignant tumours, between *in-situ *and invasive cancers and can be useful to assess oestrogen receptor status. This study was carried out to assess the reliability of core biopsy in predicting the grade and type of cancer accurately as obtaining this information can influence initial therapeutic decisions.

**Patients and methods:**

A total of 105 patients who had invasive breast carcinoma diagnosed by core biopsy in year 2001 and who subsequently underwent surgical management were included. The core biopsy results were compared with final histology with the help of *kappa *statastics.

**Results:**

A moderate level of agreement between the predicted grades and final grades was noted (kappa = 0.585). The agreement was good between predicted and final type of tumour (kappa = 0.639).

**Conclusions:**

Core biopsy as a predictor of grade and type has limited use at present. We suggest that initial clinical decisions should not be based on the results of core biopsy.

## Background

Core biopsy is rapidly replacing fine needle aspiration cytology (FNAC) as a procedure of choice for the triple assessment of the breast problems. Where there is access to an experienced cytopathologist, the FNAC can provide a rapid and cost effective means of triage of patients who would benefit from more expensive core biopsy [1]. Core biopsy is, however, more reliable predictor of the pathology [2-4] and can distinguish between benign and malignant tumours and between *in-situ *and invasive cancers. Collins *et al *have shown that majority (83%) of core biopsies and excisional procedures demonstrate exact histological agreement [5]. Core biopsy may give a good guide to grade and type of the cancer and it can also be used to assess the oestrogen receptor (ER) status. Core biopsy has also been found to be a good tool to assess effect of neo-adjuvant chemotherapy on the grade of breast cancer [6].

As the range of options for the treatment of the breast cancer widens, it has become increasingly important that clinicians are provided with accurate prognostic information to base the initial therapeutic decisions on. Prognostic factors for breast cancer have been extensively studied. Histological grade and type can be used to predict biological behaviour as has been assessed by overall survival and local recurrence for women with primary breast carcinoma [7-9]. Histological grade is one of the three prognostic factors used in calculating the Nottingham Prognostic Index [10].

The aim of this study, therefore, was to see how reliable core biopsy is in predicting the grade and the type of cancers, as that could influence the further management of breast cancer.

## Patients and methods

All patients with invasive breast cancer diagnosed by the core biopsy and treated subsequently by surgical excision in the year 2001, at a district general hospital were included in the study. Of the 105 patients whose records were studied retrospectively, 47 lesions were palpable and 58 lesions were screen detected. The core biopsies were performed under ultrasound guidance as a part of triple assessment and at least four cores were obtained from palpable lesions and six or more from screen detected lesions with a 22 mm automated core biopsy gun. Two dedicated breast pathologists had authorised all the reports. Age of patients ranged from 35 to 84 with a median age of 62 years. The histology reports for the core biopsy and final histology were extracted and compared. Carcinoma *in-situ *diagnosed by core biopsy and patients who underwent neo-adjuvant therapy were excluded from the study. Level of agreement between core and excision biopsy was assessed using *kappa *statistics.

## Results

Of 105 patients there was no prediction of grade in 2 patients and in 19, a prediction of grade 1 or 2 or grade 2 or 3 was made. This left 84 where a clear prediction was made. On final histology 35 (33.3%) were categorised as grade I, 45 (42.8%) were grade II and 25 (23.8%) were grade III. The predicted grades versus final grade results are detailed in table 1.

**Table 1:** Cross tabulation showing predicted verses final grade

	Final grade			
	***Grade***	***1***	***2***	***3***
Predicted grade	***1***	21	0	0
	***2***	5	35	9
	***3***	1	6	7
	***1 or 2***	7	1	1
	***2 or 3***	0	2	8
	***Not predicted***	1	1	0

Kappa = 0.585

Of the 84 cores in which clear prediction of grade was made 63 (75%) were correct. All 21 of grade 1's were predicted correctly, 35 (71%) of grade 2's were predicted correctly but only 7 (50%) or grade 3's were predicted correctly on core biopsy. Of the predicted grade 2's which were reclassified, 5 (10%) were downgraded and 9 (18%) were upgraded. Of the reclassified grade 3's, 6 (43%) were downgraded to grade 2 and 1 (7%) was downgraded to grade 1.

Of 105 patients, 101 patients had a prediction of type made. Of 84 cases predicted to be ductal, 81 (96%) were correct and one case was reclassified as mixed histology. Of the 14 predicted to be lobular 9 (64%) were correct and one reclassified as mixed (Table 2). Of the three cases predicted as mixed only one was mixed on final pathology.

**Table 2:** Cross tabulation of predicted verses final tumour types.

	**Final type**			
	*Type*	***Lobular***	***Ductal***	***Ducto-lobular***
**Predicted type**	***Lobular***	9	4	1
	***Ductal***	2	81	1
	***Ducto-lobular***	1	1	1
	***Uncertain***	1	2	1

Kappa = 0.639

In general the level of agreement between the predicted grades and final grades was moderate (kappa = 0.585) and between predicted and final types was slightly better (kappa = 0.639).

## Discussion

Fajardo *et al *reported percutaneous, image guided biopsy to be an accurate diagnostic alternative to surgical biopsy in women with mammographically detected suspicious breast lesions [11]. The false negative results occur to a lesser degree with image guided core biopsy [12]. However needle size [13] or amount of clinical material obtained [14] has not been found to influence the histology results. A recent study has shown that access to expert breast pathologists can avoid inconsistencies observed in the category of borderline lesions between the expert and general pathologists [15].

Histological grade and type, tumour size and presence or absence of axillary node metastases is well-recognised prognostic factors of breast cancer. Tumour grade, size and nodal involvement are three factors considered in Nottingham Prognostic Index [10]. Histological grade and type on their own can be helpful in predicting the biological behaviour of the tumour as regards to local recurrence and overall survival [7-9]. Preoperative grading and typing with core biopsy, therefore, can influence further management of the cancer this is all the more important as the sensitivity and specificity of mammogram for predicting grade or type is poor [16].

Green Hough (1925) was the first to categorise the breast tumours into three grades according to its differentiation. He also assessed the association of grades with "cure" though the term cure was not clearly defined [17]. Since then a clear association between grades and prognosis has been established [17-23]. Higher the grade, greater is the chance of the tumour relapsing [24,25]. It has also been noted that oestrogen receptor (ER) negative tumours are usually of higher grade [26-28]. Higher the tumour grade more aggressive is the tumour and nodal involvement too is directly related to aggressiveness of the tumour [29]. All these factors suggest that higher the grade of tumour more radically should it be managed. Knowing the grade accurately, preoperatively, would help in planning out further management of the tumour. It is possible to identify all these prognostic factor in core biopsy. A small earlier study has shown 80% sensitivity of core biopsy for correct diagnosis and a poor (50%) sensitivity for diagnosing invasive cancers in mammographically detected cancers [30]. It is not possible to comment on this in present study as only invasive cancers were included in the present study.

Of the two major histological types, lobular is known for its multifocality and multicentricity and its diffusely infiltrating nature [31]. It is important to correctly identify lobular carcinoma, as these tumours are often hormone responsive [21].

Our results suggest that the prediction of grade and type of breast cancer from core biopsy has only limited use at present. The group of patients we would like to be predicted most accurately would have been those with a high-grade and lobular type, for the reasons stated above. Our results suggest that these patients are most difficult to predict in practice. However, present study being retrospective has its own drawbacks. A prospective study specifically aimed at Kappa statistics between core biopsy and final histopathology may be able to answer this question better. Further refinements are needed in technique of core biopsy and these technical innovations will ultimately improve the results of core biopsy.

## Competing interest

The author(s) declare that they have no competing interests.

## Authors' contributions

**AD**: Original idea, planning of study, background search, data compilation and drafting the manuscript.

**TG**: Data collection, data compilation, help with the manuscript drafting.

**SH**: Overall supervision and guidance with the study, helped in the analysis and helped with manuscript drafting and revisions.

All authors read and approved the final version

## Funding source

None
